# Trends in access to water supply and sanitation in 31 major sub-Saharan African cities: an analysis of DHS data from 2000 to 2012

**DOI:** 10.1186/1471-2458-14-208

**Published:** 2014-02-28

**Authors:** Mike R Hopewell, Jay P Graham

**Affiliations:** 1 School of Public Health and Health Services, Department of Environmental and Occupational Health, George Washington University, 2100 M St. NW, Ste. 203 M, Washington, DC 20037, USA

**Keywords:** Water supply, Sanitation, Sub-Saharan Africa, Open defecation, Water collection time

## Abstract

**Background:**

By 2050, sub-Saharan Africa’s (SSA) urban population is expected to grow from 414 million to over 1.2 billion. This growth will likely increase challenges to municipalities attempting to provide access to water supply and sanitation (WS&S). This study aims to characterize trends in access to WS&S in SSA cities and identify factors affecting those trends.

**Methods:**

DHS data collected between 2000 and 2012 were used for this analysis of thirty-one cities in SSA. Four categories of household access to WS&S were studied using data from demographic and health surveys – these included: 1) household access to an improved water supply, 2) household’s time spent collecting water, 3) household access to improved sanitation, and 4) households reporting to engage in open defecation. An exploratory analysis of these measures was then conducted to assess the relationship of access to several independent variables.

**Results:**

Among the 31 cities, there was wide variability in coverage levels and trends in coverage with respect to the four categories of access. The majority of cities were found to be increasing access in the categories of improved water supply and improved sanitation (65% and 83% of cities, respectively), while fewer were making progress in reducing the amount of time spent collecting water and reducing open defecation (50% and 38% of cities, respectively). Additionally, the prevalence of open defecation in study cities was found to be, on average, increasing.

**Conclusions:**

Based on DHS data, cities appeared to be making the most progress in gaining access to WS&S along metrics which reflect specified targets of the Millennium Development Goals. Nearly half of the cities, however, did not make progress in reducing open defecation or the time spent collecting water. This may reflect that the MDGs have led to a focus on “improved” services while other measures, potentially more relevant to the extreme poor, are being neglected. This study highlights the need to better characterize access, beyond definitions of improved and unimproved, as well as the need to target resources to cities where changes in WS&S access have stalled, or in some cases regressed.

## Background

It has been estimated that sub-Saharan Africa’s (SSA) urban population will nearly triple by 2050, increasing from 414 million to over 1.2 billion [1]. This growth of urban populations in SSA has already begun to affect progress towards the Millennium Development Goals, which aim to increase access to improved water supply and sanitation (WS&S). Between 1990 and 2010, there was no change in access to improved sanitation in urban SSA, which remained at 43% of the population [2]. During this same time, there was similar stagnation seen in access to water supply in the urban areas of SSA. Overall, access to improved water supply increased in SSA from 49% to 61% between 1990 and 2010. In urban areas, however, access remained at 83% over the same period. Additionally, those in urban SSA with access to piped water into their homes or yards declined from 43% to 34% from 1990 to 2010 [2].

Due in large part to rapid urban population growth, the number of urban SSA residents using the worst forms of sanitation or water supply has increased between 1990 and 2010. In that time, the number of people using untreated surface water as their primary source of water grew from 5 to 9 million, and those whose primary sanitation method was open defecation increased from 16 to 25 million [3]. This is especially troubling as WS&S may be more critical in low-income, urban communities where population density is high and infectious diseases can more easily spread. A cross-sectional study of children in the Republic of Congo found that the odds of diarrhea among urban children was 3.5 times that of rural children [4]. There is also limited evidence that improving sanitation in densely populated urban informal settlements – where the human fecal waste may also be more densely concentrated – can have a greater impact on diarrhea and soil-transmitted helminths (STHs), and subsequently nutrition and mortality, than in less densely populated communities [5,6].

It is estimated that most urban population growth in SSA will occur in informal settlements or slums. Informal settlements are difficult to define and there is substantial diversity in conditions between and within cities. Typified by congestion and poor access to vital resources, such as WS&S, residents of these areas often see health outcomes much worse than non-slum dwelling urban residents and even rural inhabitants [7]. A 2006 study in Kenya found great disparities in the prevalence of diarrheal disease between residents of informal settlements and the rest of the country. The prevalence of diarrheal disease for children under the age of 3 was 11% in slums, compared to 3% in both rural areas and the country as a whole [8].

The urban poor of SSA, who are often residents of informal settlements, have much lower rates of access to both WS&S. Sixty four percent of the poorest quintile of urban residents have access to improved water supply compared to 94% of the wealthiest quintile. Similar disparities are seen in sanitation with 42% of the poorest quintile using improved or shared sanitation compared to 91% of their wealthy counterparts [2]. Furthermore, research has documented that improving sewage infrastructure in urban settings can greatly improve health outcomes [9-11].

Given these rapid rates of urbanization and stagnating rates of access to WS&S in urban areas in SSA, further examination of this area is critical. Identifying the current levels of access in individual cities of SSA, in which direction they are heading, and identifying the driving factors will become more important as cities grow larger and struggle to provide these basic services to their residents. The aims of this study were to 1) estimate the average annual change in access to WS&S in the largest cities across SSA from 2000-2012; 2) explore how city-level and country-level factors may affect progress towards increasing access to these basic WS&S services; and 3) identify and discuss the limitations of measuring trends using demographic and health survey data.

## Methods

### Data sources

Publicly available demographic and health survey (DHS) data for Sub-Saharan Africa were used to conduct this research. All surveys included in this analysis were conducted from the year 2000 and later. DHS are nationally-representative household surveys where standard surveys are usually conducted every three to five years. These surveys provide information on various topics including housing characteristics and household health. The Measure DHS program implements a standard model questionnaire approach in order to collect data that is comparable across countries (DHS Questionnaires 2012). Each country has surveyed anywhere from 5,000 to 30,000 households in each survey. The sample is representative at a national, residence, and regional level. For cities, the sampling frame is defined by that country’s census bureau. Sampling is typically based on a stratified two-stage cluster design. The first stage uses census files to identify enumeration areas (EA), small administrative units with defined boundaries and a known population size. Most surveys select 300-500 EAs with probability proportional to population size. In the second stage, an updated listing of households in each selected EA is used from which sample households are drawn. Data are collected by a team consisting of six to eight field workers who travel to conduct interviews and enter data onto either paper questionnaires or electronic files on the computer (DHS Methodology 2012).

### City selection criteria

Selection criteria for cities to be included in the study were: 1) a population size greater than 1 million defined by UN:DESA; 2) to be located in a low- or lower-middle income country of SSA as defined by The World Bank; and 3) DHS data sets were available for two time points within 2000-2012 [see Additional file 1: Table S3]. Given these criteria, the study included 31 cities from 20 countries.

### Dependent variables

The study included four measures of access, which were estimated based on demographic and health surveys (DHS) for each country:

• Percent of households with access to an improved water supply

• Percent of households with access to improved sanitation

• Percent of households that spend 30 minutes or more collecting water

• Percent of households reporting to engage in open defecation

In selecting these measures of access to WS&S, two indicators were selected to mirror the development benchmarks of improved access set by the MDGs. The DHS surveys indicate drinking water source and type of sanitation for each household. Data from the DHS were categorized as improved or unimproved based on WHO/UNICEF Joint Monitoring Programme definitions of improved water supply and sanitation [12]. The final two dependent variables (% of households reporting open defecation and reported time spent collecting water) were selected to explore access for households facing the most extreme conditions in the study cities. Data on open defecation were available in the same question of the DHS survey that asked about the households’ type of sanitation. Time spent collecting water was a separate question in the DHS that asked, “How long does it take to go there [to the household’s water source], get water, and come back?”. Research has suggested that if total travel time to collect water is greater than 30 minutes, households tend to collect less than the 15 to 25 liters per day that is needed to meet basic human needs [13].

To further explore access to WS&S, each access variable was assessed and analyzed in two ways. First, the most recent estimate of the percentage of households with access was determined using DHS data from the most recent year for each city. Second, the annual change in the percent of households with access was calculated using a line equation based on the available DHS data within the study period (2000-2012).

### Independent variables

In developing independent variables to conduct an exploratory analysis, the literature was reviewed to understand which factors could potentially be linked with access to WS&S. The independent variables in this study can be categorized as demographic, socio-economic, political, and environmental, and all the data used were publicly available [see Additional file 2]. The following variables were included for exploratory analysis to understand their effect in improving access to WS&S in the largest cities of SSA: 1) total city population; 2) urbanization rate; 3) population density; 4) city level GINI coefficients of inequality; 5) national per capita GDP; 6) national per capita GDP growth rate; 7) average education level of city population; 8) official development assistance for urban WS&S; and 9) flood risk. The variables have been defined and described in Table S1 [see Additional file 2].

### Population and urbanization

City population statistics and urbanization rates were obtained from the UN Department of Economic and Social Affairs’ World Urbanization Prospects, The 2011 Revision. Every two years since 1988, UN:DESA has released revised estimates of the urban and rural populations of every country and their major urban agglomerations. As data are collected on urban agglomerations of 750,000 people and greater in the UN:DESA report; all the cities included in this study have data available [1]. City density, measured in population per km^2^, was obtained from Demographia World Urban Areas 2012 [14].

### Income inequality

GINI coefficients, which consist of a mixture of income and consumption based measures, were used to measure the city-level income inequality. GINI coefficients were obtained from UN Habitat’s Global Urban Indicators Database [15]. The Global Urban Indicators contained GINI coefficients for only 22 of the 31 cities included in this analysis.

### City wealth

A quantitative indice of wealth for the cities analyzed was not available. Similar to other studies, such as *Jacobsen et al.*[16], national level data were used as proxy measures to estimate the per capita GDP and per capita GDP growth at the city level [16] – these were obtained from the World Bank [17].

### Education

Data for the average level of education were obtained from each city’s most recent DHS. The measure for education was the percentage of heads of household in each city that completed secondary school.

### External funding for urban WS&S

Official Development Assistance (ODA) for water supply and sanitation, measured in millions of dollars per year averaged over the study period of 2000-2012, was obtained from the Organization for Economic Co-operation and Development (OECD). While unable to parse out data for the individual cities of this study or even to the urban level nationally, this study used data on funds allocated to “water supply and sanitation – large systems” as a proxy for ODA in urban areas. Large systems are defined by OECD for water supply as: potable water treatment plants, intake works, storage, water supply pumping stations, and large scale transmission/conveyance and distribution systems; and for sanitation as: large scale sewerage including trunk sewers and sewage pumping stations, domestic and industrial wastewater treatment plants [18]. To compare countries of varying sizes, this urban WS&S ODA proxy for each country was divided by the urban population of each country, giving a per capita assistance variable.

### Flooding

Data on flood occurrence were obtained from the World Research Institute’s Aqueduct Global Water Risk Mapping Tool [19]. The tool categorized cities into four levels of risk based on the number of floods they experienced between 1985 and 2011: Low (0-1), Medium (2-3), High (4-9), and Very High (9+).

There are important limitations to using these indices for the analysis. Some of the data used for our independent variables were national level indices (e.g. per capita GDP), while the WS&S access measures were city-level. Further, it was unclear where the official development assistance for WS&S was targeted and whether the cities in our analysis received support. Given the limitations of the exposures of interest, the analysis should be considered exploratory in nature only.

### Statistical analysis

The development of descriptive statistics for WS&S access – city-level prevalence of open defecation or the average annual percentage point change in open defecation, for example – were the main focus of the analysis. Exploratory data analysis using simple linear regression was also conducted to look at the associations between the independent variables and the four measures of WS&S access. Independent variables were first analyzed individually with the annual change of each WS&S access indicator, or dependent variable, using a bivariate linear regression model. Multivariate regression analysis was also conducted to control for the other independent variables in measuring association. Of the 31 cities that met the initial selection criteria, several were omitted from certain categories of the assessment [see Additional file 2].

## Results

In assessing the four measures of access to WS&S through DHS data, significant variability was observed among the study cities. There was also substantial variability in how cities progressed in their ranking across the four categories. Some cities were found in similar relative position across categories, for example, while others were near the top in some categories and near the bottom in others.

Major differences among the cities were observed for the average annual change in access among the study’s four categories of access. The majority of cities were found to be making at least minimal progress in the categories of improved water supply and improved sanitation (65% and 83% of study cities, respectively), while fewer of the study cities were found to be making progress in time spent collecting water and open defecation (50% and 38%, respectively).

Based on the DHS data, 3 of the 31 cities were either making progress or approaching universal coverage across all four measures of access: Lagos, Dakar, and Douala. A further 8 cities made progress in three of the measures of access: Nairobi, Dar es Salaam, Ouagadougou, Bamako, Conakry, Kampala, Benin City, and Kumasi.

None of the study cities were found to be regressing across all four measures of access to WS&S, and only three were found to be regressing in three of the four: Abuja, Kaduna, and Harare.

### Improved water supply

**Figure 1:**
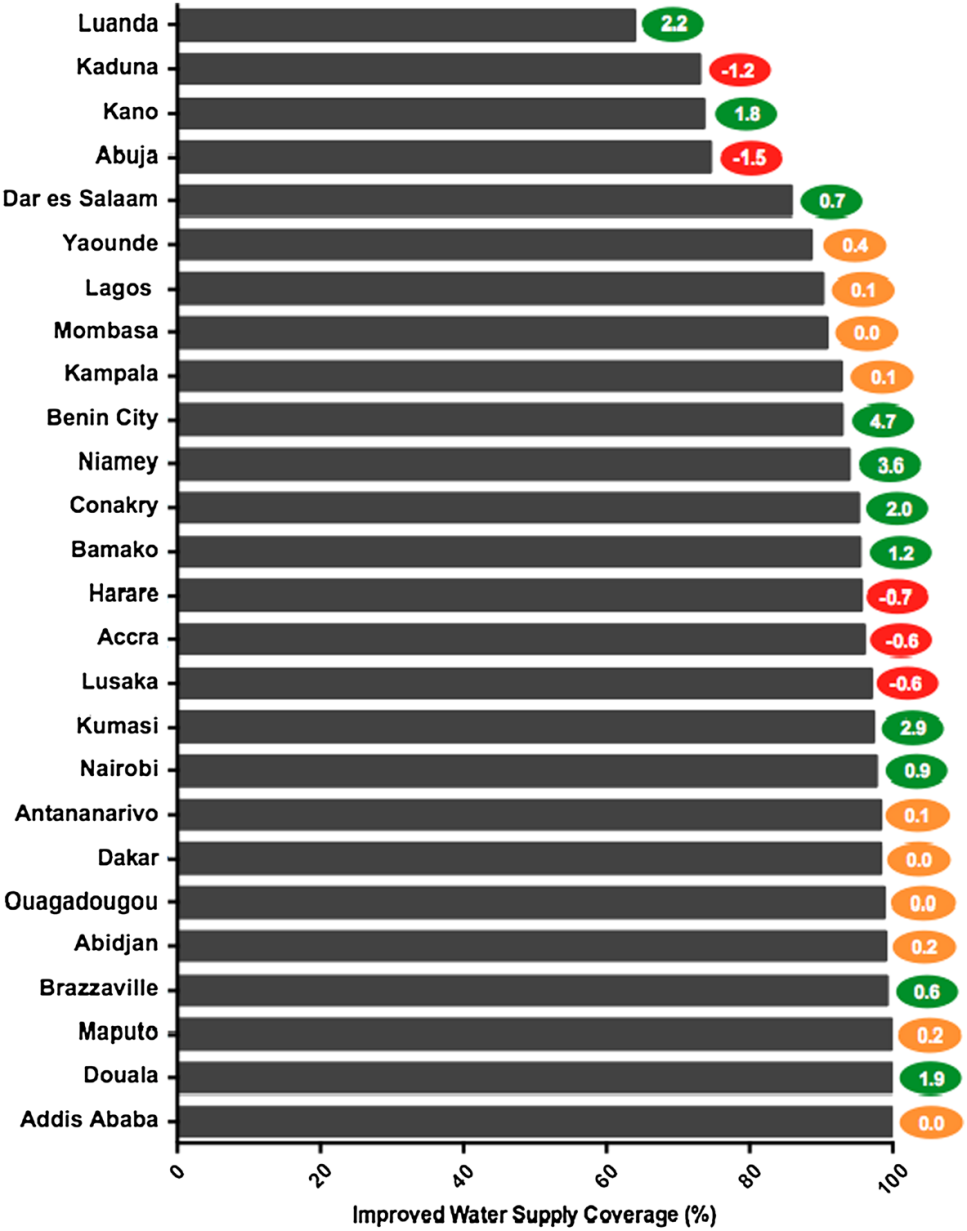
**Coverage Levels for Improved Water Supply.** Figure 1 illustrates the most current levels of coverage and annual rate of change for improved water supply for the study cities. The cities are ranked bottom to top from most desirable access to poorest access. The colored oval on the right of each bar indicates the annual rate of change, measured in percentage points, for each city. The color of the oval indicates positive (green), negative (red), or minimal change (yellow). See Table S2 for complete data.

Of the cities analyzed, 26 had sufficient DHS data to calculate the most recent coverage levels for improved water supply during the study period of 2000-2012. The average coverage level for improved water supply for the 26 cities was found to be 91.7%. As shown in Figure 1, this figure ranged from the poorest coverage of Luanda with 63.9% to Addis Ababa with 99.9%. Like Addis Ababa, many other cities were approaching universal coverage for improved water supply. Fifteen cities were found to have coverage levels of at least 95%: Addis Ababa, Douala, Maputo, Brazzaville, Abidjan, Ouagadougou, Dakar, Antananarivo, Nairobi, Kumasi, Lusaka, Accra, Harare, Bamako, and Conakry. An additional five cities had coverage levels higher than 90%: Niamey, Benin City, Kampala, Mombasa, and Lagos. Of the remaining six cities, only two had coverage levels of more than 80% (Yaoundé and Dar es Salaam), three above 70% (Abuja, Kano, and Kaduna), and finally Luanda with the poorest coverage (63.9%). Luanda’s poor coverage is largely due to a sizeable percentage of their population that receives their water via tanker truck, nearly 35%.

As a whole, the 26 cities were generally increasing access to water supply according to the data, with an average improvement of 0.7 percentage points annually during the study period. With many cities approaching 100% coverage, their rates of change were more likely to be close to zero as they neared this ceiling. Even with that mitigating factor, there remained a wide range of cities’ average annual change. The city with the greatest progress over the study period was Benin City with an average annual improvement of 4.7 percentage points. Conversely, Abuja’s coverage level was found to be declining the fastest, decreasing 1.7 percentage points annually. In addition to Abuja, four more cities were found to have decreasing coverage levels: Kaduna, Harare, Accra, and Lusaka. Additionally alarming were Abuja and Kaduna’s regressive trends combined with already low coverage levels, both in the bottom four cities for most recent improved water coverage levels.

Ten cities had coverage levels that were making minimal or no progress in either direction (between -0.5 and +0.5 annual percentage points): Yaoundé, Lagos, Mombasa, Kampala, Antananarivo, Dakar, Ouagadougou, Abidjan, Maputo, and Douala. While preferable to regressive trends, these stagnating coverage levels in addition to population growth mean that the actual number of people without access to improved water supply is increasing in many of these cities. The remaining ten cities, as well as the aforementioned Benin City, made significant progress during the study period (>0.5 annual percentage points): Luanda, Kano, Dar es Salaam, Niamey, Conakry, Bamako, Kumasi, Nairobi, Brazzaville, and Douala.

### Time spent collecting drinking water

**Figure 2:**
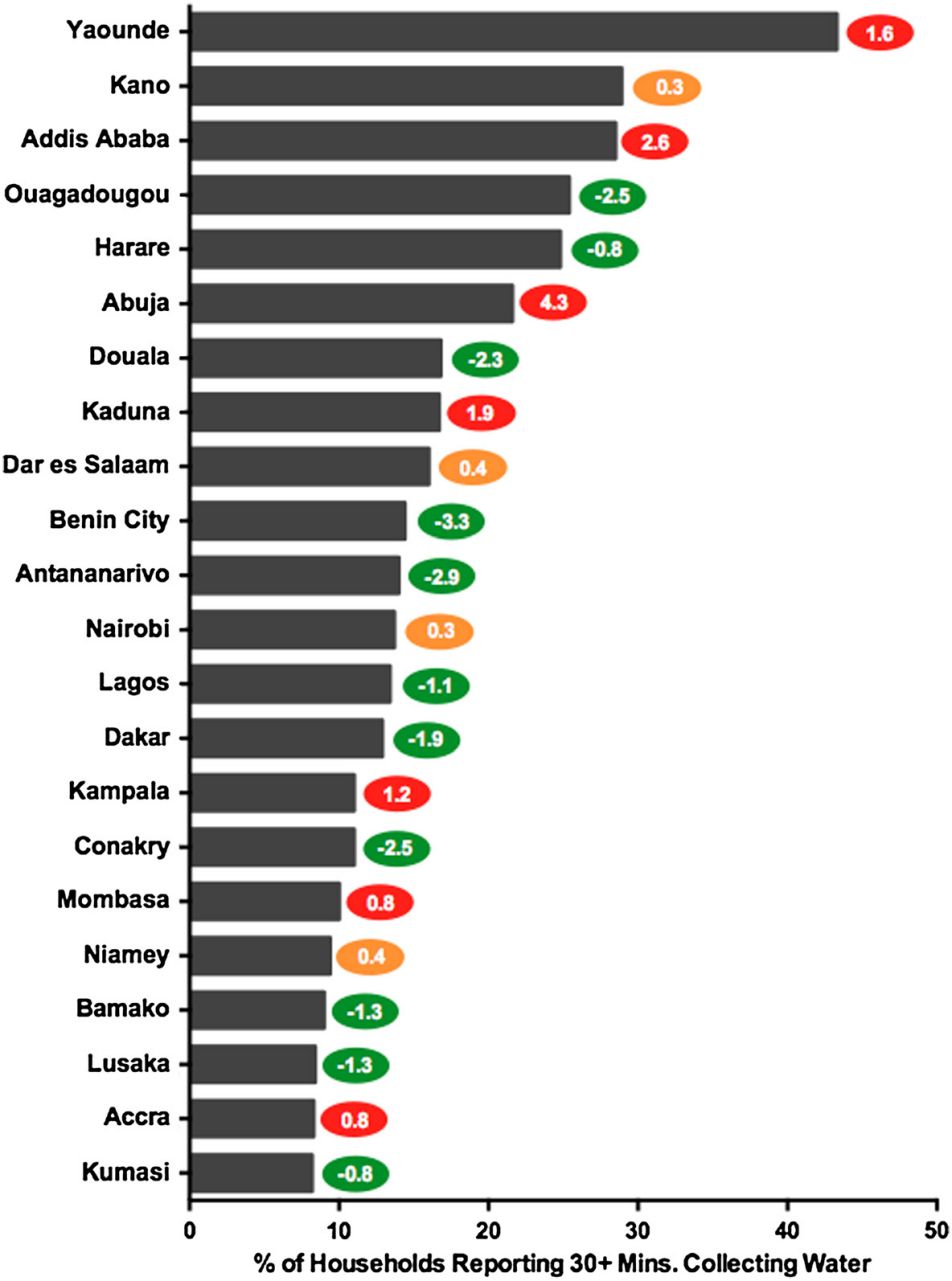
**Time Spent Collecting Water.** Figure 2 illustrates the most current measures of time spent collecting water (the proportion of households spending 30 or more minutes collecting water) and the annual rate of change of this measure for the study cities. The cities are ranked bottom to top from most desirable access to poorest access. The colored oval on the right of each bar indicates the annual rate of change, measured in percentage points, for each city. The color of the oval indicates positive (green), negative (red), or minimal change (yellow). See Table S2 for complete data.

To further measure the access to water supply in the study cities, the amount of time households reported collecting water was analyzed. Of the study cities, sufficient data were available for 22 of the cities. Of those 22 cities, the data indicated that the average proportion of households reporting 30 or more minutes to collect water was 16.6%, but there was again a wide range seen between the cities. Shown in Figure 2, DHS data showed that as few as 8.2% of households reported spending 30 minutes or more collecting water in Kumasi, while a striking 43.3% did so in Yaoundé. This proportion was less than 15% in 12 additional cities: Accra, Lusaka, Bamako, Niamey, Mombasa, Conakry, Kampala, Dakar, Lagos, Nairobi, Antananarivo, and Benin City. Eight cities had 15% to 30% spending 30 minutes or more collecting water: Dar es Salaam, Kaduna, Douala, Abuja, Harare, Ouagadougou, Addis Ababa, and Kano. After Kano, there then exists a 14% gap before Yaoundé’s proportion of 43.3%.

In addition to Yaoundé having the greatest proportion of households reporting 30 or more minutes collecting water, this proportion was found to have increased at an annual rate of 1.6 percentage points during the study period. There were six other cities that saw significant annual increases (>0.5 annual percentage points) in this measure: Abuja, Addis Ababa, Kaduna, Kampala, Mombasa, and Accra with Abuja’s 4.3 percentage point increase the most dramatic. Four cities made no significant change over the study period: Kano, Dar es Salaam, Nairobi, and Niamey. Half of the 22 cities saw decreases in this collection-time measure over the course of the study period: Ouagadougou, Harare, Douala, Benin City, Antananarivo, Lagos, Dakar, Conakry, Bamako, Lusaka, and Kumasi. This many cities heading in the right direction might indicate overall progress in this measure, but the average change in proportion for the 22 cities was just -0.1 annual percentage points.

When looking at the two measures of water access together, there are some interesting results. Addis Ababa, Douala, and Ouagadougou had some of the strongest coverage levels of improved water supply, but all ranked as some of the lowest performing cities when looking at the measure for collection-time. It appears in these locations that although households are using better sources of water, that they are having to travel farther or wait longer in lines to utilize them.

### Improved sanitation

**Figure 3:**
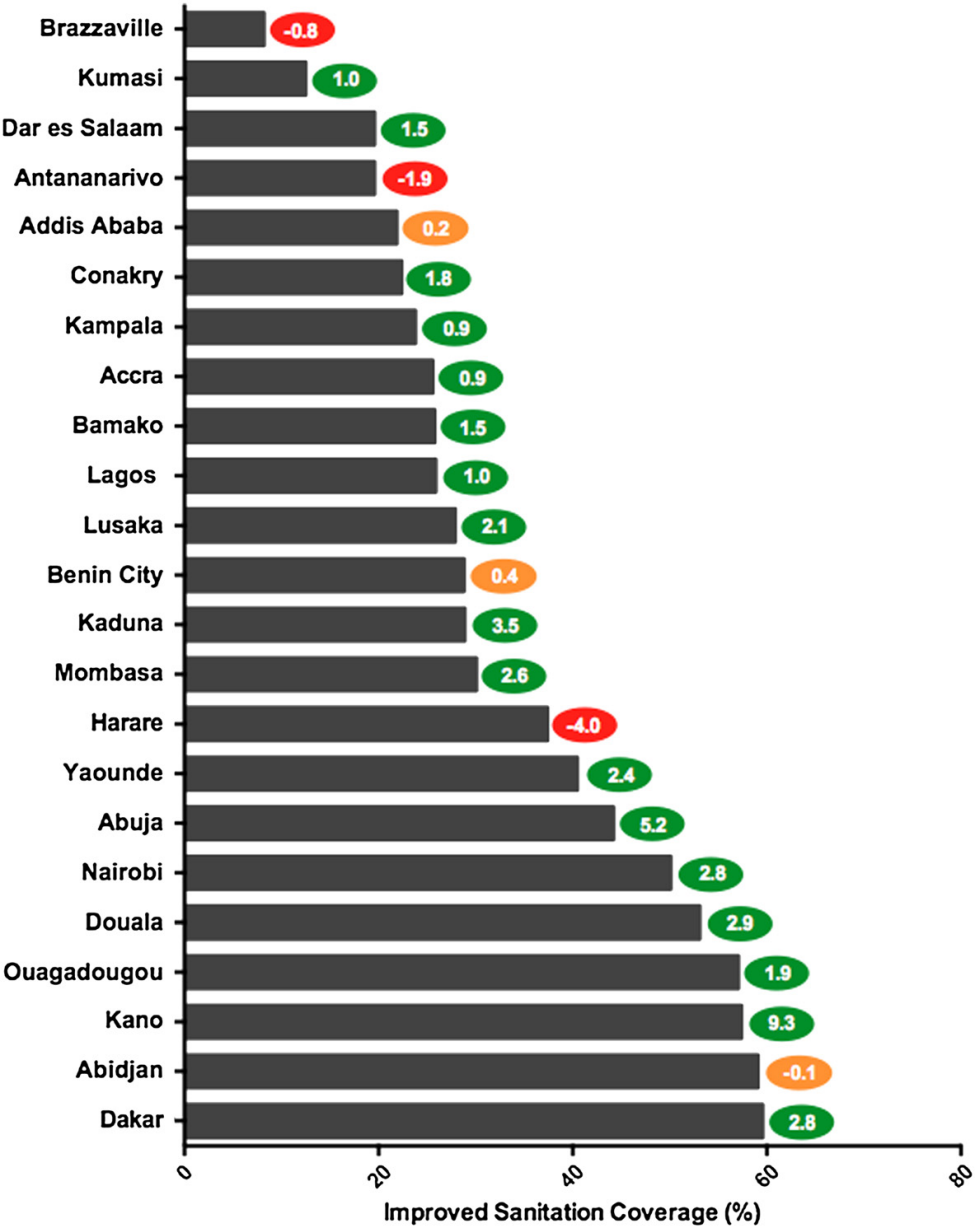
**Coverage Levels for Improved Sanitation.** Figure 3 illustrates the most current levels of coverage and annual rate of change for improved sanitation for the study cities. The cities are ranked bottom to top from most desirable access to poorest access. The colored oval on the right of each bar indicates the annual rate of change, measured in percentage points, for each city. The color of the oval indicates positive (green), negative (red), or minimal change (yellow). See Table S2 for complete data.

Shown in Figure 3, 23 of the study cities had sufficient data on improved sanitation. Similar to global trends, these cities had much lower coverage levels for improved sanitation than they did for water supply. Based on DHS data, the average improved sanitation coverage for the cities was 32.4%. Even the highest performer, Dakar, had only 59.6% of its households using improved facilities. Five additional cities were above 50%: Abidjan, Kano, Ouagadougou, Douala, and Nairobi. Ten cities’ coverage levels were between 50% and 25%: Abuja, Yaoundé, Harare, Mombasa, Kaduna, Benin City, Lusaka, Lagos, Bamako, and Accra. The cities with the poorest improved sanitation coverage, below 25%, were Kampala, Conakry, Addis Ababa, Antananarivo, Dar es Salaam, Kumasi, and Brazzaville with the lowest coverage of 8.2%.

Although overall coverage levels for improved sanitation were relatively low for the 23 cities, the data indicate that they are making good progress. The average annual percentage point change across the study period was an increase of 1.6%. Additionally, only three cities’ coverage level regressed significantly (less than -0.5 percentage points): Brazzaville, Antananarivo, and Harare. Three cities were found to have no significant change (between -0.5 and 0.5): Addis Ababa, Benin City, and Abidjan. As stated in regards to water supply, these stagnating coverage levels are preferable to regression, but combined with population growth lead to a growing population of people without access to improved sanitation facilities.

The data suggest that 17 cities made significant progress in increasing coverage levels of improved sanitation during the study period. Some cities made significant progress; Kano and Abuja had annual percentage point increases of 9.3% and 5.2%, respectively. Seven other cities increased by more than 2 percentage points each year: Lusaka, Kaduna, Mombasa, Yaoundé, Nairobi, Douala, and Dakar. The remaining eight cities’ coverage levels grew between 0.5 and 2 percentage points annually during the study: Kumasi, Dar es Salaam, Conakry, Kampala, Accra, Bamako, Lagos, and Ouagadougou.

### Open defecation

Reducing the worst form of sanitation, open defecation, is a vital step in improving sanitation in cities. Prevalence of open defecation provides a measure of sanitation access that highlights the poorest and most vulnerable city dwellers.

Of the study cities, 26 cities had sufficient data to calculate prevalence of open defecation and annual rates of change. Typically seen in rural areas, prevalence of open defecation was fairly low in the 26 cities, with an average of 3.4% households reporting the practice in the most recent DHS surveys. Two cities, Dar es Salaam and Maputo, had no households reporting open defecation in the most recent survey, and a further ten cities had a prevalence that was below 1%: Nairobi, Abidjan, Luanda, Conakry, Dakar, Bamako, Harare, Douala, Yaoundé, and Kampala. Eight more cities had rates below 3%: Antananarivo, Kano, Accra, Lusaka, Mombasa, Ouagadougou, Lagos, and Brazzaville. Prevalence of open defecation takes an alarming upturn in the remaining six cities: Kumasi (3.7%), Addis Ababa (5.8%), Kaduna (9.2%), Niamey (10.7%), Abuja (16.0%), and Benin City (22.9%).

**Figure 4:**
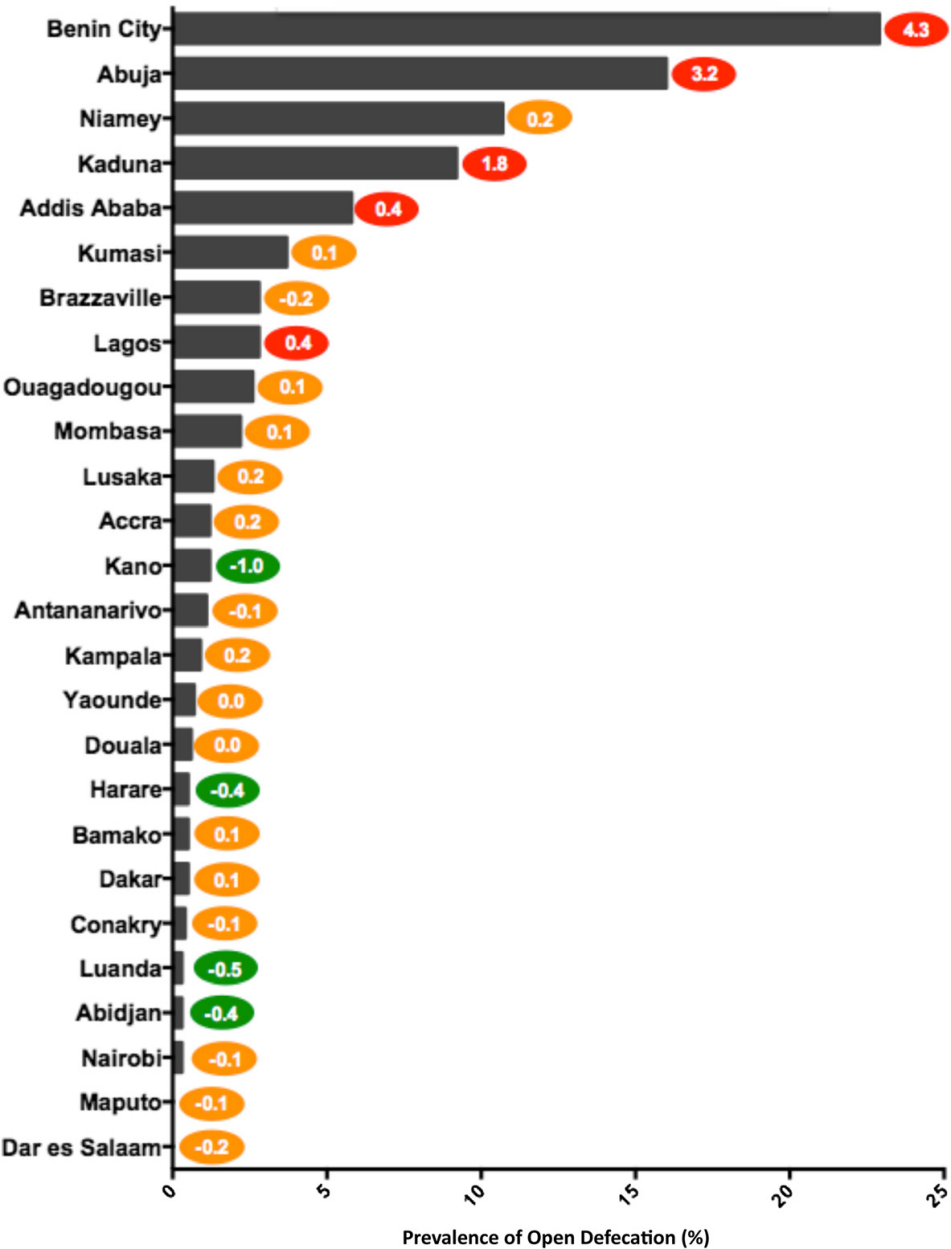
**Prevalence of Open Defecation.** Figure 4 illustrates the most current prevalence and annual rate of change of open defecation for each of the study cities. The cities are ranked bottom to top from most desirable access to poorest access. The colored oval on the right of each bar indicates the annual rate of change, measured in percentage points, for each city. The color of the oval indicates positive (green), negative (red), or minimal change (yellow). See Table S2 for complete data.

Unlike the progress seen in coverage of improved sanitation, the study cities’ prevalence of open defecation is actually increasing. According to the data, the average annual percentage point increase for the 26 cities was 0.3%. As shown in Figure 4, only 4 cities made significant progress in decreasing prevalence of open defecation (less than -0.3% annual percentage point change): Kano, Harare, Luanda, and Abidjan. Many of the cities that are approaching 0% open defecation are unable to make significant progress, but the cities with more room for improvement are heading in the wrong direction. Of the eight cities with the highest prevalence of open defecation, none made significant reductions and five of the eight saw significant increases (Lagos, Addis Ababa, Kaduna, Abuja, Benin City). Abuja and Benin City possess the highest levels of open defecation (16% and 22.9%, respectively) as well as the most quickly increasing levels (3.2 and 4.3 annual percentage points, respectively).

### Findings of bivariate and multivariate analyses

The exploratory data analysis portion of this study was limited to a select number of factors, and was merely an exploratory look into which factors are associated with progress on WS&S in the largest cities of SSA.

Across the nine independent variables, little significance was found with any of the measures of access to WS&S. No independent variable was found significantly correlated with more than one measure of access, and the majority (7 independent variables) were found not to be significantly correlated (at a 90% confidence level or higher) with any of the measures of WS&S access.

The three factors that were found to be significantly associated with any of the measures of access were city population density, national level GDP growth, and official development assistance for water supply and sanitation, large systems. It was found that denser cities were associated with higher annual increases in access to improved sanitation during the study period. Cities with higher national level GDP growth were associated with greater improvements of improved sanitation. Finally, a reduction in the prevalence of open defecation was associated with higher levels of ODA for WS&S.

Data for all four measures of access and the independent variables are available in the summary table (see Additional file 3: Table S2).

## Discussion

Given the large number of cities analyzed, this study provides only a broad snapshot of the issues. This method, however, could easily be applied with more depth for any given city.

### Progress in WS&S access

It was found that of the four categories of access to WS&S, the study cities were making the most progress in improved water supply and improved sanitation, the measures mirroring the benchmarks set by the MDGs, while half or fewer of the cities were making progress in the other two measures. This possibly indicates that the MDGs have focused efforts on this improved/unimproved variable while other issues, such as open defecation or time spent collecting water, are being neglected.

When assessing improved sanitation and open defecation in these cities, it appears there is a growing disparity. In many of these cities, both access to improved sanitation and the percentage of households practicing open defecation were increasing. This was most apparent when looking at Abuja, a city that was found to have a relatively high coverage level of improved sanitation and one that was increasing at over 5 percentage points each year during the study period. Conversely, the city was found to have the second highest prevalence of open defecation, and this prevalence is growing at 3.2 percentage points annually. This might indicate that sanitation improvements were not available to the poorest and most marginalized citizens of Abuja. It is intriguing to compare Abuja to Dar es Salaam, a city with one of the lowest coverage levels for improved sanitation. This coverage though, is increasing significantly at 1.5 percentage points annually and no households in Dar es Salaam reported practicing open defecation in the most recent survey. Dar es Salaam seems to be increasing access to sanitation in a more equitable way than Abuja. Though this study did not do so, it would be interesting to look at these trends (improved water supply & open defecation) by wealth quintile. Such an analysis could perhaps shed light on the role wealth might play in this disparity.

### Population density

The results linking population density to average annual change in sanitation coverage suggested that, perhaps contrary to what might be expected, the most densely populated cities are making the greatest progress in increasing coverage levels of improved sanitation.

### Gross domestic product and official development assistance

The analysis of economic growth variables yielded some interesting results. While economic growth was unquestionably a vital part of governments ability to provide essential services to their people, these results did little to reinforce the notion that either national level GDP or GDP growth leads directly to increased access to WS&S in large cities, particularly in water supply, time collecting water, or open defecation. The results of this analysis indicated that GDP growth may be more critical in providing sanitation than in the other measures of access. ODA, as with all the independent variables of the study, was also found to have an insignificant relationship across three of the four measures of access, only being found positively correlated with decreases in the prevalence of open defecation. What was most striking about this finding was that the proxy of “large scale WS&S systems” (sewerage systems, wastewater treatment plants, etc.) used for development assistance targeting urban WS&S would not necessarily be expected to affect households practicing open defecation. One hypothesis for this unexpected correlation involves perhaps the more obscure effects that ODA may have. While the assistance itself, in a monetary sense, might not reach those populations practicing open defecation, the resources can create WS&S-related job positions and bring WS&S to the fore politically. This was an interesting finding given that ODA is often considered ineffective, and that access to these vital services come naturally from economic growth.

### DHS limitations

While DHS and other nationally representative surveys are likely the most useful data for comparing cities in terms of WS&S access, it is important to highlight their limitations. One major limitation was how DHS may fail to include populations living in informal settlements. If the boundary of the sampling frame does not include peri-urban informal settlements, for example, then these communities will be underrepresented. This limitation likely varies from city to city and restricts the conclusions that can be drawn when comparing cities in this analysis. Additionally, for each of these cities, informal populations are rapidly changing (most often increasing), which has an effect on the accuracy of the trends that can be assessed for each city from year to year. Furthermore, as DHS looks to improve its surveying and how to better include these marginalized populations, future surveys will lose comparability with previous ones. A feature of the DHS surveys that was not utilized by this study was the GPS data that accompanies the household surveys. A thorough geospatial analysis accompanying the analysis of this study would likely have led to a better understanding of what, if any, populations of each city were underrepresented.

### Water resources

In analyzing factors that drive access to WS&S, particularly water supply, it might be argued that water resources are a major constraint. While there is an obvious link between domestic water supply and water resources, evidence suggests that domestic water use represents a minor fraction of water withdrawals [20]. Additionally, in a study of over 100 countries, when controlling for per-capita income, the proportion of urban households in countries classified as “water stressed” with improved water supplies was no less than the proportions seen in non “water stressed” countries [21].

### Shared sanitation facilities

Much debate continues as to whether shared or public toilets can be considered improved sanitation. The WHO/UNICEF Joint Monitoring Programme, for the purposes of the MDGs, classifies any shared or public sanitation facility as unimproved, and therefore this study did the same. Due to an inability to measure actual use or the hygiene and safety of shared sanitation facilities, the JMP currently considers all these facilities unimproved, even though most shared facilities may use an improved technology [22]. Studies have shown that the more users a facility has, the less hygienic it is likely to be, but also that toilet facilities shared by four or fewer households, can be considered “acceptable or improved” [23].

For this analysis, the use of shared facilities varied greatly between the study cities. In classifying every household that used shared facilities as having unimproved access, there were likely shared facilities that would meet improved hygienic and safety criteria, but were still classified as unimproved. The use of shared sanitation facilities is on the rise in developing countries, and while the JMP’s dichotomous classification of improved/unimproved eases measurement, it is currently and will only further become an incomplete measure.

### Difficulties of measuring and defining access

Identification of who has access to improved WS&S is a complex process. In this estimation, many factors of access are not taken into account. This is particularly true for water supply for which measures of water quality, quantity, cost, seasonal changes in access are not taken into account by the DHS survey. Failure to account for these aspects likely overestimates access to what might be considered a sufficient water supply, particularly for urban residents.

Studies have shown that although many urban households may be classified as having ‘improved’ access to drinking water, the quality and quantity of this water remains poor or inadequate. In densely populated urban areas, a household may be close enough to use a standpipe as their source of water (a piece of hardware classified as ‘improved’), however, they may share that standpipe with dozens of other households, limiting the amount of water truly available to them [24].

The results of this study underscore the complexity of attempting to define access to both water supply and sanitation. The need to create dichotomous variables of improved and unimproved WS&S can be useful in creating development goals such as the MDGs, but in doing so, the subtleties of access are lost.

With the current MDGs set to expire in 2015, there is a need to incorporate the many aspects of WS&S access into the measures used in the post-2015 development agenda. For water supply these aspects include quality, quantity, cost, time spent collecting water, and seasonal fluctuations. For sanitation, along with identifying shared facilities that might be adequate, issues of cost, distance from the home, and seasonality need to be considered as well.

### Strengths and Limitations

The primary limitations to this study were the lack of reliable city-level data and the use of independent variables, of varying quality, from different data sources than the dependent variables. Different data sets measure cities differently (e.g. differing geographic borders, etc.), which can limit the conclusions drawn from the findings.

Due to inadequate city-level data, this study explored how national level indices could affect access. Rather than omit important variables such as GDP, GDP growth rates and official development assistance, national level data were used as proxy measures for city-level economic conditions. This use of national-level data as proxies to estimate city-level data has been used in other studies dealing with the same dearth of city-level data [16].

For each city included in this study, only two surveys within the 12-year study period were used to assess progress in access to WS&S. If the surveys were conducted early in the decade, then the changes to access observed may not represent the city’s most recent progress. The difference in when the demographic and health surveys were conducted for each country, shown in Table S3, would likely affect the changes in access observed for that particular city.

Finally, while this study examined the largest cities of sub-Saharan Africa, these cities are not a complete representation of “urban sub-Saharan Africa”. In the entire continent of Africa (including Northern Africa), cities of more than one million people only accounted for 31.6% of Africa’s total urban population in 2010. Additionally, of the 63.8 million-person increase in the urban population of Africa between 2005-2010, only 27% of that was in cities of over one million [25].

Despite these limitations, this study had several strengths. Previous reports, such as the UN HABITAT’s State of African Cities 2010 Report and the World Bank’s Future of Water in African Cities report, have presented city level data on access to water supply and sanitation. The World Bank report analyzed individual cities along 45 different criteria [16]. This study aimed to build upon these prior reports and included trend data in the measure of access to WS&S, for a more complete picture of the progress or regress in these cities. It was also the goal of this study to clearly display where cities’ levels of access are in comparison to each other as well as the direction each city is heading. Similar to prior research, this study analyzed access to WS&S against several factors. Beyond the work done previously, this study quantitatively analyzed these factors and explored any possible statistical significance.

## Conclusion

There was substantial variability between the cities studied in the levels of access within each of the four measures of access. Variability of access was also seen within cities themselves across the four categories of access. Major differences among the cities were also observed for the average annual change in access among the study’s four categories of access. The majority of cities were found to be making at least minimal progress in the categories of improved water supply and improved sanitation, while fewer of the study cities were found to be making progress in time spent collecting water and open defecation.

As shown in Figures 1 and 4, many of the study cities seemed well positioned and heading in a direction to achieve universal coverage of improved water supply and on track to eliminate open defecation, at least within the cities as defined by that country’s census bureau. Few cities, however, were approaching universal coverage of improved sanitation.

Given the exploratory nature of the analysis of driving factors, it was difficult to draw conclusions about how the different factors included in this analysis affected access to WS&S. More effort is needed to understand the factors that influence access to WS&S in cities of low-income countries. As national surveys, such as the DHS, improve their methods over time, they will be able to better characterize marginalized urban inhabitants, and better be able to assess access levels as well as trends. Improved DHS data coupled with reliable city-level data for other factors, will allow for more rigorous statistical analysis to hypothesize the drivers of access.

As cities continue to grow in number and size throughout sub-Saharan Africa, it will become increasingly important to identify trends in access to WS&S and the potential driving factors. This understanding will allow officials to more effectively target resources. For example, local municipalities, as well as international development agencies, could use this knowledge to tailor strategies to address negative trends in accordance with the specific circumstances of each city. It will also be important that the multiple levels of access – not just ‘improved’ and ‘unimproved’ access – be considered to ensure that a more complete characterization of access is determined. Finally, improving our understanding of city-level performance in terms of driving factors and access trends could facilitate the exchange of knowledge and sharing of best practices between cities and may result in more effective investment of external funds, including official development assistance and concessionary loans.

## Abbreviations

AMCOW: African minister’s council on water; DHS: Demographic & health survey; FWAC: Future of water in Africa (Report); GDP: Gross domestic product; JMP: Joint monitoring programme; MDGs: Millennium development goals; OD: Open defecation; ODA: Official development assistance; OECD: Organization for Economic Co-operation and Development; SSA: Sub-Saharan Africa; STHs: Soil transmitted helminths; UN: United Nations; UN:DESA: United Nations Department of Economic and Social Affairs; UNICEF: United Nations Children's Fund; WASH: Water, sanitation and hygiene; WHO: World Health Organization; WRI: World Research Institute; WS&S: Water supply and sanitation.

## Competing interests

The authors declare that they have no competing interests.

## Authors’ contributions

MH and JG collaborated in designing the study. MH conducted the data analysis and drafting of the paper. JG offered support in data analysis and edited several iterations of the paper. Both authors read and approved the final manuscript.

## Pre-publication history

The pre-publication history for this paper can be accessed here:

http://www.biomedcentral.com/1471-2458/14/208/prepub

## Supplementary Material

Additional file 1: Table S3 DHS datasets used for each city included in the study. Click here for file

Additional file 2 Supplemental materials. Click here for file

Additional file 3: Table S2 Summary of data used for the analysis. Click here for file
