# Supplementary material for: Trends in access to water supply and sanitation in 31 major sub-Saharan African cities: an analysis of DHS data from 2000 to 2012
Source: BMC Public Health. 2014 Feb 28;14:208. doi: 10.1186/1471-2458-14-208 (PMC3942065; doi:10.1186/1471-2458-14-208)
Supplement: Additional file 1: Table S3 — DHS datasets used for each city included in the study. [file 1471-2458-14-208-S1.pdf]

Table 3. DHS datasets used for each city included in the study.

City	Data Set Used														
	1998	1999	2000	2001	2002	2003	2004	2005	2006	2007	2008	2009	2010	2011	2012
Lagos, Nigeria						DHS - IV					DHS - V				
Kinshasa, DRC										DHS - V					
Luanda, Angola										DHS - IV				DHS - V	
Abidjan, Cote d'Ivoire		DHS - III						DHS - V							
Nairobi, Kenya						DHS - IV							DHS - VI		
Kano, Nigeria						DHS - IV					DHS - V				
Dar es Salaam, Tanzania								DHS - IV							DHS - VI
Addis Ababa, Ethiopia		DHS - IV												DHS - VI	
Dakar, Senegal								DHS - IV						DHS - VI	
Ibadan, Nigeria						DHS - IV					DHS - V				
Accra, Ghana						DHS - IV					DHS - V				
Douala, Comeroon							DHS - IV							DHS - V	
Abuja, Nigeria						DHS - IV					DHS - V				
Ouagadougou, Burkina Faso						DHS - IV							DHS - VI		
Antananarivo, Madagascar							DHS - IV					DHS - V			
Kumasi, Ghana						DHS - IV					DHS - V				
Yaounde, Cameroon							DHS - IV							DHS - V	
Bamako, Mali				DHS - IV									DHS - VI		
Maputo, Mozambique						DHS - IV								DHS - VI	
Conakry, Guinea		DHS - IV						DHS - V							
Harare, Zimbabwe									DHS - V					DHS - VI	
Kampala, Uganda				DHS - IV										DHS - VI	
Kaduna, Nigeria						DHS - IV					DHS - V				
Lumbumbashi, DRC										DHS - V					
Mbuji-Mayi, DRC										DHS - V					
Lusaka, Zambia					DHS - IV					DHS - V					
Brazzaville, Congo								DHS - V				DHS - V			
Benin City, Nigeria						DHS - IV					DHS - V				
Niamey, Niger	DHS - III						DHS - V								
Ogbomosho, Nigeria						DHS - IV					DHS - V				
Mombasa, Kenya						DHS - IV							DHS - VI		
