# Supplementary material for: Trends in access to water supply and sanitation in 31 major sub-Saharan African cities: an analysis of DHS data from 2000 to 2012
Source: BMC Public Health. 2014 Feb 28;14:208. doi: 10.1186/1471-2458-14-208 (PMC3942065; doi:10.1186/1471-2458-14-208)
Supplement: Additional file 2 — Supplemental materials. [file 1471-2458-14-208-S2.docx]

**Supplemental Material**

**Trends in access to water supply and sanitation in 31 major sub-Saharan African cities: an analysis of DHS data from 2000 to 2012**

Mike R. Hopewell and Jay P. Graham

**Selection of independent variables**

**Population size.** There is the potential that as the population of cities grow, municipalities will be increasingly challenged to provide basic services to WS&S. Large populations can be a hindrance to governments providing adequate WS&S, particularly when coupled with other factors. Conversely, large populations can also be drivers of GDP and can provide a wealth of human capital [11].

**Urban growth and population density.** With SSA cities’ populations growing at staggering rates, increased population densities for these cities is likely inevitable. The effects of these more crowded conditions on access to WS&S are important to consider. As the report indicates, density measures should be considered reasonable approximations due to the fact that estimates of land area and population (used to calculate density) are not done at precisely the same time. Average density gives a general measure of population concentration in these cities, but it is important to note that they do not capture variations of population density within these cities.

**GINI coefficient.** In urban areas, it is generally the poorest and most marginalized populations that lack access to WS&S. Therefore, it was hypothesized that cities with greater inequality would have poorer access to these resources.

**GDP and GDP growth.** Of all the variables included in this study, it was expected that variables of financial development of these cities, per capita GDP and per capita GDP growth, would be the best predictors of access to water supply and sanitation.

**Education.** Several studies have highlighted the link between education and access to WS&S at the household level. This study explored whether this relationship would be seen at a city level. For the study, education was measured as the proportion of households where the head of household completed secondary school. While data were retrieved from DHS household surveys, this study assessed this variable at city averages rather than at the household level.

**Official Development Assistance and Assistance for urban WS&S.** It was expected that a relationship would exist between foreign assistance and access to WS&S, but it was unclear if it would be significant, given the number of cities analyzed, and which WS&S access variables it would affect. Given the classifications of OECD data, the “large systems” measure gave a reasonable indication of the amount of assistance given to urban areas, but the limitations of the measure should be noted as well. OECD warns that urban water supply and sanitation projects are typically “large system”, but can also be “basic” [17].

**Flooding.** Environmental factors can undoubtedly play a large role in access to WS&S. This study analyzed the occurrence of floods in the study cities in relation to the dependent variables of WS&S access. None of the study cities met the criteria for low risk, six cities were medium risk, twelve high risk, and fourteen very high risk. Given the current and future effects of climate change, identifying the cities where access to these vital resources was most affected by flooding could prove important.

**Missing factors.** In determining independent variables, there were several factors that were hypothesized to have significance, but could not be included in this quantitative study for one of many reasons. While very likely a significant factor, political will was determined to be too subjective to include. The presence of water supply or sanitation policies was considered for inclusion, however, these data were found to be limited at the city level and there is a lack of consensus on what policy measures at the municipal level could affect access. The level of conflict in cities was also considered for inclusion as an independent variable. Difficult to quantify and with limited data on city-level conflict, this factor was omitted. Another variable that was omitted was the percentage of urban dwellers living in informal settlements. While cities with larger slum populations would likely be an important factor in a city’s access to WS&S, access to these resources is how informal settlements are often defined, which would bias any analysis of this variable.

**Description of Dependent and independent variables**

Table 1. Dependent and Independent Variables Description

| **Independent Variable** | **Variable Description** |
| --- | --- |
| Total City Population (Continuous) | 2010 estimate of city population from UN:DESA |
| Urbanization Rate (Continuous) | Annual percentage growth calculated from 2000-2010 change population from UN:DESA |
| Average City Density (Continuous) | 2012 estimated population/km^2^ via Demographia |
| City Level Gini Coefficient (Continuous) | A mixture of income and consumption Gini coefficients measuring economic inequality for each city from UN HABITAT’s Global Urban Indicators Database |
| National Per Capita GDP (Continuous) | 2011 estimated GDP via World Bank |
| National Per Capita GDP Growth Rate (Continuous) | Average annual GDP growth rate between 2000 and 2011 via World Bank |
| Official Donor Assistance for Water Supply and Sanitation (Continuous) | Average national-level ODA for water supply and sanitation – large systems between 2000  and 2010 via OECD |
| Education (Continuous) | Percentage of heads-of-households that have completed secondary school |
| Flooding Occurrence 1985 – 2011 (Categorical) | 1 – Low (0-1) |
|  | 2 – Medium (2-3) |
|  | 3 – High (4-9) |
|  | 4 – Very High (9 or more) |
| **Dependent Variables** | **Variable Description** |
| Improved Access to Water Supply (Continuous) | - Most recent estimate of access calculated from DHS - Avg. annual rate of change calculated from DHS between 2000 – 2012. |
| Improved Access to Sanitation Facilities (Continuous) |  |
| Percent of Households Spending 30+ Mins. Collecting Water (Continuous) |  |
| Percent of Individuals Practicing Open Defecation (Continuous) |  |

***Omission of Selected Categories for Several Cities***

*Ibadan and Ogbomosho, Nigeria*

Due to their geographic proximity and to the regions which DHS included in their surveys, data for these two cities were unable to be differentiated in assessing WS&S indicators. Both cities are located inside the Oya state of Nigeria and no smaller regional criterion was included in the survey. Therefore, each city was omitted from all aspects of the analysis.

*Kinshasa, Lubumbashi, and Mbuji-Mayi, Democratic Republic of Congo*

DHS data were only available for 2007 for the Democratic Republic of Congo. No trend data could be calculated for these three cities, so they were omitted from the assessment. The available data for these cities were included in the summary table excluding water collection time, which was not collected.

*Abijan, Cote d’Ivoire; Luanda, Angola; Maputo, Mozambique; Brazzaville, Congo*

The DHS surveys for the countries of these four cities did not collect information on water collection time. Consequently, they were not included in the analysis for this access variable.

*Luanda, Angola*

In the Angola DHS surveys, information on whether sanitation facilities were shared or not was not collected. Without this information it was impossible to identify those that had improved sanitation, so Luanda was omitted from the assessment for the sanitation indicator.

*Maputo, Mozambique*

In Mozambique’s DHS surveys, there was no differentiation between improved and traditional latrines, meaning a proper figure of those who had access to improved sanitation could not be calculated. For this reason Maputo was omitted from the assessment for the sanitation indicator.

*Niamey, Niger*

In the older of the two Niger DHS surveys used to calculate trend data for Niamey, there was no differentiation between improved and traditional latrines. Without being able to properly identify those that had improved access to sanitation, Niamey was omitted from the assessment for the sanitation indicator.
