# Supplementary material for: Trends in access to water supply and sanitation in 31 major sub-Saharan African cities: an analysis of DHS data from 2000 to 2012
Source: BMC Public Health. 2014 Feb 28;14:208. doi: 10.1186/1471-2458-14-208 (PMC3942065; doi:10.1186/1471-2458-14-208)
Supplement: Additional file 3: Table S2 — Summary of data used for the analysis. [file 1471-2458-14-208-S3.pdf]

Table 2. Summary of data used for the analysis

City	Improved WS		Water Collection Time		Improved San		OD Rates		City-Level Population Indices			National-Level GDP		City-Level Education	Per Capita ODA for Large Scale WS & S	City-Level Flood Risk
	Coverage Level (%)	Change (% per Year)	30+ Mins Collection (%)	Change (% per Year)	Coverage Level (%)	Change (% per Year)	OD Prevalence (%)	Change (% per Year)	2010 Pop. (x1000	Pop. Growth (% per year)	Pop. Density (People/km^2)	2011 Per Capita GDP (US$)	2000-2011 Annual Growth	Heads of Household Completing Secondary School		
Lagos, Nigeria	90.2	0.14	13.4	-1.10	25.9	1.02	2.8	-0.21	10578	4.5	12700	1502	6.3	75.17	0.35	Very High
Kinshasa, DRC	93.8	-	-	-	13.5	-	1.3	-	8754	6.2	15500	231	4.2	83.3	1.88	High
Luanda, Angola	63.9	2.15	-	-	~	~	0.3	-0.01	4772	8.4	10600	5318	9.9	-	0.15	High
Abidjan, Cote d'Ivoire	99.0	0.15	-	-	59.1	-0.13	0.3	-0.40	4125	3.6	13500	1195	0.2	-	1.04	High
Nairobi, Kenya	97.7	0.93	13.7	0.37	50.1	2.84	0.3	-0.46	3523	5.9	7500	808	4	76	4.57	Very High
Kano, Nigeria	73.6	1.82	28.9	0.09	57.4	9.31	1.2	0.24	3395	3.0	15700	1502	6.3	45.03	0.35	Very High
Dar es Salaam, Tanzania	85.8	0.68	16.0	0.54	19.6	1.49	0.0	-0.05	3349	4.3	7100	532	6.7	30.09	5.24	High
Addis Ababa, Ethiopia	99.9	0.02	28.5	2.59	21.9	0.18	5.8	0.36	2930	2.3	9200	357	8.3	28.89	1.80	Very High
Dakar, Senegal	98.3	0.00	12.9	-1.87	59.6	2.78	0.5	-0.16	2863	4.1	14900	1119	4.1	36.04	5.74	High
Ibadan, Nigeria	~	~	~	~	~	~	~	~	2837	2.7	8400	1502	6.3	-	0.35	Very High
Accra, Ghana	96.0	-0.63	8.3	0.75	25.6	0.94	1.2	-0.95	2342	4.0	4100	1570	6.3	74.04	2.25	Very High
Douala, Comeroon	99.9	1.94	16.8	-2.28	53.1	2.88	0.6	-0.03	2125	4.3	12700	1260	3.4	69.26	2.17	Moderate
Abuja, Nigeria	74.5	-1.46	21.6	4.32	44.2	5.20	16.0	3.20	1995	13.9	10000	1502	6.3	79.69	0.35	High
Ouagadougou, Burkina Faso	98.8	0.04	25.4	-2.24	57.1	1.94	2.6	0.07	1909	10.7	6000	600	5.7	37.21	5.26	Very High
Antananarivo, Madagascar	98.3	1.98	14.0	0.28	19.6	-1.86	1.1	-0.31	1879	2.9	9100	465	2.8	72.54	0.64	Very High
Kumasi, Ghana	97.3	2.87	8.2	-0.78	12.5	0.97	3.7	0.05	1834	5.5	6500	1570	6.3	69.04	2.25	High
Yaounde, Cameroon	88.6	0.41	43.3	1.58	40.5	2.44	0.7	-0.03	1801	3.3	12600	1260	3.4	63.86	2.18	Moderate
Bamako, Mali	95.4	1.20	9.0	-1.25	25.8	1.48	0.5	0.07	1699	4.9	6800	669	5.2	9.88	2.57	High
Maputo, Mozambique	99.8	0.20	-	-	~	~	0.0	-0.18	1655	6.2	7400	535	7.2	31	5.37	High
Conakry, Guinea	95.2	0.02	11.0	1.20	22.4	1.83	0.4	-0.09	1653	3.5	11000	498	2.8	40.19	1.29	Moderate
Harare, Zimbabwe	95.6	-0.69	24.8	-0.86	37.4	-4.01	0.5	0.06	1632	1.8	2700	747	-3.3	81.83	0.30	High
Kampala, Uganda	92.8	0.13	11.0	-2.45	23.8	0.92	0.9	0.17	1598	4.6	3500	487	7	70.64	4.90	Very High
Kaduna, Nigeria	73.0	-1.21	16.7	1.91	28.9	3.45	9.2	1.84	1561	3.2	10200	1502	6.3	59.68	0.35	Very High
Lumbumbashi, DRC	87.2	-	-	-	18.5	-	7.0	-	1543	6.1	10500	231	4.2	67.72	1.88	Moderate
Mbuji-Mayi, DRC	90.6	-	-	-	31.8	-	2.7	-	1488	6.7	11700	231	4.2	63.51	1.88	High
Lusaka, Zambia	97.0	-0.64	8.4	-1.26	27.9	2.10	1.3	0.17	1451	3.5	8200	1425	5.4	63.97	3.54	Very High
Brazzaville, Congo	99.2	0.61	-	-	8.2	-0.80	2.8	0.35	1323	2.9	10540	3485	4.8	80.95	2.20	Moderate
Benin City, Nigeria	92.9	4.67	14.4	-3.33	28.8	0.44	22.9	4.27	1302	3.4	6100	1502	6.3	68.32	0.35	Moderate
Niamey, Niger	93.9	3.60	9.4	-0.44	~	~	10.7	0.18	1048	5.4	8900	374	3.8	33.22	3.43	Very High
Ogbomosho, Nigeria	~	~	~	~	~	~	~	~	1032	2.9	7600	1502	6.3	-	0.35	Very High
Mombasa, Kenya	90.8	0.00	10.0	1.11	30.1	2.60	2.2	0.00	1003	4.7	11900	808	4	64.72	4.57	Very High
